# Optimization of virus-mediated functional imaging based on quantitative evaluation of transduction efficiency in pigeon (*Columba livia domestica*)

**DOI:** 10.1016/j.psj.2025.105961

**Published:** 2025-10-08

**Authors:** Zhengyue Zhou, Yezhong Tang, Wenbo Wang, Xin Yang, Zihan Zhuang, Tianmei Pu, Feng Jiang, Zhendong Dai

**Affiliations:** a School of Artificial Intelligence/School of Future Technology, Nanjing University of Information Science and Technology, Nanjing Jiangsu 210044, China; b Chengdu Institute of Biology, Chinese Academy of Sciences, No.9 Section 4, Renmin Nan Road, Chengdu, Sichuan 610041, China; c Institute of Bio-inspired Structure and Surface Engineering, Nanjing University of Aeronautics and Astronautics, Nanjing, Jiangsu 210000, China; d Brain Case Biotechnology Co., Ltd, China; e College of Electrical, Energy and Power Engineering, Yangzhou University, Yangzhou Jiangsu 225000, China

**Keywords:** Poultry, Telencephalon, Mesencephalon, Promoter, Serotype

## Abstract

Currently, virus-mediated functional imaging integrated with optogenetic techniques has been used successfully in both neuroanatomical track tracing and accurate regulation of animal's social and cognitive behaviors. In avian, only a few studies focused on the mesencephalon and brainstem, compared to those on the telencephalon and diencephalon. To widen adeno-associated viruses (AAV) in avian studies, we tested the transduction efficiency of different types of sera and promoters and designed a Python script to analyze quantitatively the fluorescence intensity of brain slices. Our experiments on pigeons consisted of the serotypes for anterograde and retrograde labeling, promoters for virus gene transcription, injection doses and periods for transduction, and different optogenetic components. We found serotypes 1 and 11 with ubiquitous promoters transducing well at all brain regions, AAV transduction being more efficient in the telencephalon than mesencephalon and the hChR2 component significantly affecting transduction efficiency. The optimum injection dose and transduction period guideline for each location and serotype were determined. Our findings can be refereed during the related study and will aid future research in avian.

## Graphical abstract

**Figure:**
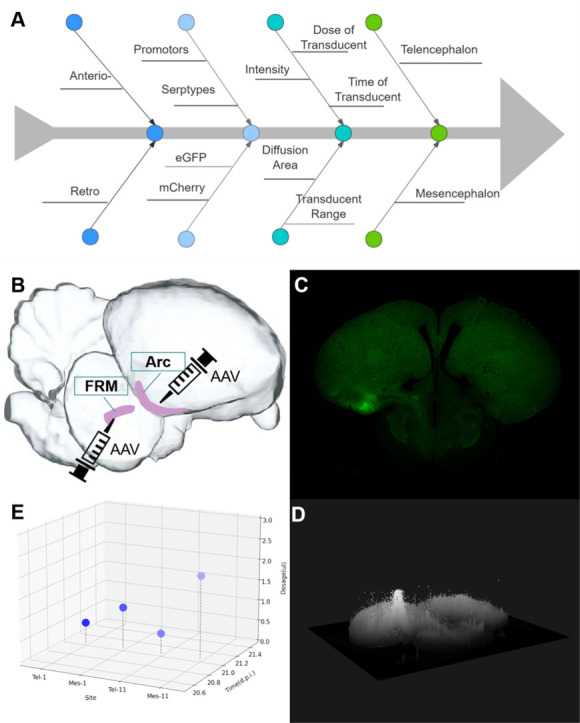

## Introsuction

Adeno-associated viruses (AAV) as vectors are extensively used in neural studies for their wide array of promoters that are compatible with various tissues or cell types and for their numerous serotypes suitable for both afferent and efferent tracing purposes (Chen et al., 2020; Kojima et al., 2024; Lin, 2020). These vectors were designed and constructed as probes to elucidate specific cellular functions or as gene therapy (Griffin et al., 2019; Pignataro et al., 2017) agents for treatment (Norrman et al., 2010; Xu et al., 2019). Additionally, virus-mediated functional imaging and optogenetic techniques could precisely regulate animal social and cognitive behaviors at the cellular level *in vivo* (van der Zouwen et al., 2021; Gatto and Goulding, 2018; Islam et al., 2020). For instance, the movement pace and locomotion gait of mice could be modulated using optogenetic protocols targeted at the cuneiform nucleus (Dautan et al., 2021). Strategies using Cre or FLP would precisely activate or suppress downstream neurons from specific circuits on upstream (Bouvier et al., 2015; Sánchez-Valpuesta et al., 2019). However, these studies often impose restrictions on the cells of mice or mammals as hosts only because they are research hotspots. This is a reciprocal process in which high research hotspots contribute to the diversity of tool vectors, and more versatile tool vectors promote the development of this field. Yet, in avian research, this collaboration is not as evident.

While some experiments have leveraged AAVs to elucidate specific neural circuit functions in avians, such as interpreting the vocal learning process in juvenile songbirds or investigating the sensitive period in adolescent zebra finches, nearly all these studies were conducted in labs with strong biological backgrounds, focusing primarily on unraveling mechanisms related to species-specific functions in bird brains (Sánchez-Valpuesta et al., 2019; Rook et al., 2021; Düring et al., 2020). Unfortunately, guidelines for selecting appropriate AAVs based on research objectives were still lacking for avian studies. Instructions on choosing viruses suitable for mammals do not directly translate to avian applications due to differences in species, brain size, and developmental periods. Therefore, this study aimed to identify anterograde and retrograde AAVs capable of successfully transduction in avian species and to determine the optimal dose and transduction period for these viruses.

The most significant factors influencing AAV transduction ability are the promoters and serotypes (Sabatino et al., 2022). Promoters control the expression of genes carried by the vector, determining where, when, and how strongly genes are expressed in target cells. They can be categorized into tissue-specific, broad-spectrum (ubiquitous), inducible, cell cycle, and viral promoters based on their functions (Xu et al., 2019; Hong et al., 2007). Additionally, serotype refers to variants of the viral capsid protein that encapsulate the viral genome, which dictate the ability to target and transduce specific cell types or tissues with specific tropism, immune evasion, and tissue penetration properties (Lin, 2020; Gao et al., 2004). According to these features, in this study, we selected three dominant broad-spectrum promoters—CMV, CAG, and EF1α—alongside serotypes 1, 2, 9, 11, and DJ (Lerch et al., 2012; Aschauer et al., 2013; Dou et al., 2021; Wang et al., 2017; Blankvoort et al., 2018; Large et al., 2021). The primary goal was to identify viruses capable of expressing both anterograde and retrograde characteristics in avian brains. We also determined the optimal dose and transduction period for viruses targeting the telencephalon and mesencephalon, respectively. This work not only lays the foundation for future avian research in fields such as optogenetics, which require tool viruses like AAVs but also serves as a reference for other studies necessitating AAV transduction in avians.

## Materials and methods

**Fig. 1:**
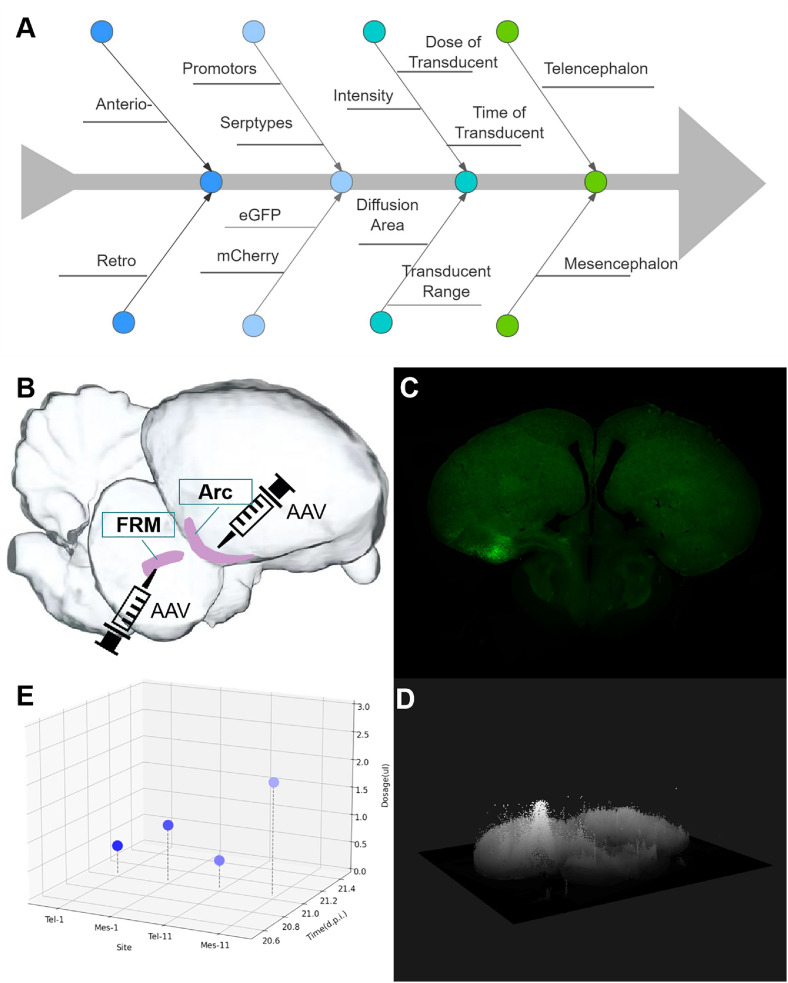
Graphical abstract of this study. A) Experimental design of treatment groups; B) Locations of primary transduction test sites in the pigeon brain; C) Representative scan results of brain slices; D) Visualization of transduction patterns using VTK analysis; E) Optimization of transduction time and viral dosage for each brain region and AAV serotype.

For this study, a series of experiments was designed to evaluate the expression efficiency of adeno-associated viruses (AAVs) in the avian brain. The transduction capacity of different viral vectors was first tested. A custom Python script was used to quantify fluorescence intensity, transduction range, and diffusion area. Viral vectors showing relatively higher efficiency were subsequently injected into different brain regions. Temporal and dose gradients were applied to determine the optimal transduction conditions for each area. The overall workflow is presented in Fig. 1A–E.

### Experiment animals

In this study, adult male and female pigeons weighing 350–450 g were used for viral transduction experiments. All surgical procedures were performed under general anesthesia (0.00135 ml/g) to minimize animal suffering. All experimental protocols were approved by the Jiangsu Association for Laboratory Animal Science (Jiangsu, China). The pigeons were housed in a dovecote under a natural light–dark cycle with access to water and cereal. Following viral injection, each pigeon was housed individually and allowed to freely hover outside the cage once per day.

### Injection *in vivo* and histology

All surgical procedures were performed under aseptic conditions. Pigeons were anesthetized with pentobarbital sodium (0.00135 ml/g) and placed in a stereotaxic apparatus (Zhou et al., 2021; Liu et al., 2017). Once the anesthesia took effect, holes were drilled into the skull at coordinates aligned with target nuclei on a standard brain map. A glass electrode (inner diameter 0.54 mm, outer diameter 1.14 mm, no inner core) was then slowly and consistently implanted into the Hp, AI, LHy, or FRM regions. Adult pigeons received stereotaxic injections of rAAV into target brain regions using a pulled glass micropipette connected to a microsyringe pump. Injections were performed at a rate of 50–100 nL/min, with a total volume of 0.5–2 μl per site. After injection, the pipette was left in place for 5–10 min to minimize backflow. Typically, a single hemisphere of each subject received only one type of AAV, with a total dose not exceeding 2 μl per pigeon. Vet-bond Tissue Adhesive was used to seal the skull openings. The surgical area was then sutured and allowed to heal. After 21 to 42 days for transduction, the pigeons were sacrificed for analysis. Initially, pigeons were perfused with phosphate-buffered saline (PBS), followed by fixation with a 4 % paraformaldehyde (PFA) solution. The brains were preserved in 4 % PFA for eight hours, submerged in a 30 % sucrose solution for 72 hours, and then sectioned into 50-µm coronal slices using a freezing microtome (Leica CM1950, Germany). These sections were examined with a research-grade whole slide scanning system (Olympus VS200, Japan) to verify the placement of fluorescent markers within the Hp, AI, LHy, or FRM nuclei. Finally, the fluorescence in these tissue sections was analyzed to assess transduction efficiency with a customer-made script.

### Fluorescence intensity analysis

All brain slices were analyzed in the Python VTK environment, where fluorescence intensity, transduction scope, and diffusion range were calculated according to references (Gouws, 2009; Taka and Srinivasan, 2011). The transduction efficiency of AAVs in the injected brain site was measured by fluorescence intensity; their capability to propagate afferent or efferent along neurons in the sagittal plane was measured by transduction scope; and their diffusion ability in the coronal plane was assessed by converting it to the area_percentage as diffusion range. Based on this, we scanned brain slices containing eGFP or mCherry fluorescence that were converted into a pixel grayscale matrix with brightness levels from 0 to 255. This process allows us to calculate the maximum intensity value for fluorescence at the injection locis. Additionally, areas of the image with values greater than 0 were considered effective areas of the brain slice, termed positive_area. Using this pixel grayscale metric, we compared each slice to the standard brain map and delineated the transduction scope.

Slices were further segmented into five regions, with the maximum value of each segment recorded in terms of max_val_1 to max_val_5. We established thresholds based on these values as per Formula (1). This threshold was then used to calculate active_area_count according to Formula (2), determining the percentage of diffusion range at the injection locis by Formula (3). Through these methods, we conducted statistical analyses to evaluate the transduction characteristics of different AAVs.

Formula (1) $bright\_offset=a^{\left((\max\_val÷150)\times 2\right)}$
Formula (2) active_area_count=max_val(1/5)+offset
Formula (3) Area=active_area_count/positive_area*100%

### Comparison of AAVs transduction efficiency in avian

To optimize virus selection for successful transduction in pigeons, we have tested various serotypes and promoters known to facilitate successful transduction. We employed broad-spectrum promoters such as CMV, CAG, and EF1α, along with the neuron-specific promoter hSyn (Dou et al., 2021; Wang et al., 2017; Kügler et al., 2003). In vector construction, the promoters CMV, CAG, and EF1α are commonly used for over-expression both *in vitro* and *in vivo* and CMV is the most frequently used commercial vector promoter for mammalian (Liu et al., 2022). These promoters are extensively applied in mouse brain and tail vein injections tailored to specific research purposes. Additionally, the hSyn promoter, derived from the human SYN1 gene, expresses the Synapsin I protein exclusively in neurons, making it ideal for neuron-specific applications. We also explored serotypes 1, 2, 9, DJ, and 11 to assess their anterograde and retrograde transduction capabilities (Lerch et al., 2012; Aschauer et al., 2013). At present, there are many AAV serotypes variants available for neural study. AAV1 and AAV2 were recovered from apes, AAV9 from African green monkeys, respectively, and AAV11 was isolated from cynomolgus monkeys (Gao et al., 2004). It has been reported that the DJ serotype has well transduction ability in avian (Düring et al., 2020). Previous, adaptation of different serotypes to different tissues has been extensively studied in mammals. Unfortunately, these researches related to promoters and serotypes were unsuitable references for avian research. For this research, we selected various AAVs as outlined in Table 1, including six anterograde and the remaining retrograde, using the aforementioned promoters and serotypes, all sourced from a native bioproduct company. Each virus was injected at the same site in the telencephalon, followed by a 21-day expression period with each virus type used repeatedly in three subjects. Subsequently, the brains were preserved, and fluorescence was analyzed using the previously described methods. Finally, one of the most efficient transduction ability viruses with anterograde or retrograde features was selected and utilized for the next research.

**Table 1:** The fluorescence intensity of transduction in pigeon telencephalon.

un-Transduction		Transduction	
Vector	Intensity	Vector	Intensity
rAAV2-hSyn-eGFP	15	rAAV1-CAG-eGFP	149
rAAVDJ-CAG-eGFP	25	rAAV1-EF1α-eGFP	149
rAAV9-EF1α-eGFP	26	rscAAV1-CMV-eGFP	149
rAAV9-CMV-eGFP	14	rAAV11-CMV-eGFP	81
rAAV9-hSyn-eGFP	25	rAAV11-EF1α-eGFP	85
rAAV9-CAG-eGFP	14	rAAV11-CAG-mCherry	76

### Efficiency transduction in different cerebral regions

In this section, we evaluated the transduction ability at different cerebral locations. Due to the evolution divergence the brains’ shape, size, and structure experience the numerous changes between mammalian and avian. For instance, the pallium region in birds has the same function as the mammalian cortex. Birds often rely on the midbrain tectum for processing visual perception, whereas mammals typically depend on the cortex (Medina, 2007). Considering this condition, we separated the pigeon brain into regions in a comparatively rough way, such as telencephalon, diencephalon, and mesencephalon, as well as the old view of the cortex (Jarvis et al., 2005). We selected representative nuclei for each brain region: the Arcopallium intermedium (AI) for the telencephalon, the Nucleus lateralis hypothalami (LHy) for the diencephalon, the Formatio reticularis medialis mesencephali (FRM) for the mesencephalon, and the Hippocampus (Hp) for the pallium. One μl of sc-AAV1-CMV-eGFP with anterograde characteristics to the AI, LHy, FRM, and Hp was administrated, with each pigeon receiving a single injection on one side of the hemisphere. Each location was tested with three subjects, except for LHy(only one subject), over a 21-day expression period. Following this, the brains were harvested and fluorescence was analyzed. Additionally, 1 μl of AAV11-EF1α-eGFP with retrograde characteristics to the AI, LHy, and FRM was administrated. Like the anterograde study, each location had three test subjects, except for LHy, followed by a 21-day expression period, with subsequent harvest and analysis of the brain fluorescence. Finally, we conducted statistical analyses using One-way ANOVA to evaluate fluorescence intensity within and between groups based on the anterograde and retrograde characteristics.

### Time gradient measurement

As model animals, C57 mice were used commonly in neurophysiology and were recommended to be sacrificed 21 days post-injection or utilized in experiments starting 14 days post-injection (Chen et al., 2020; Inagaki et al., 2022). However, for pigeons, the situation may differ due to variations in brain size and cell type (Stacho et al., 2020; Güntürkün et al., 2013). In this study, considering the characteristics of anterograde and retrograde transmission, we evaluated how the expression period of AAVs influences transduction effectiveness in the telencephalon and mesencephalon. We administered sc-AAV1-CMV-eGFP and AAV11-EF1α-eGFP to both the AI and FRM. For each virus at each site, three time gradients were established: 21 days, 30 days, and 42 days. Each combination was tested on constant three subjects. After post-injection, brains were harvested and analyzed for fluorescence. Statistical analyses using one-way ANOVA were conducted to evaluate the fluorescence intensity, transduction scope, and diffusion range differences to measure transduction efficiency between time gradients in the telencephalon and mesencephalon under conditions of anterograde and retrograde AAVs.

### Dose gradient measurement

Although some studies have recommended doses for avian optogenetics, most involve zebra finches with relatively small brains, while others focus on the cognitive functions of pigeons or crows (Sánchez-Valpuesta et al., 2019; Düring et al., 2020; Hisey et al., 2018; Xiao et al., 2018). These studies predominantly target the telencephalon due to its functional importance (Rook et al., 2021). Consequently, the optimal dose for specific brain regions remains unclear. In this phase of our research, we established three dose gradients and paired them with two types of AAVs: sc-AAV1-CMV-eGFP and AAV11-EF1α-eGFP, injected into the mesencephalon and telencephalon of pigeons, respectively. Each pigeon received a viral injection in one hemisphere, and each virus at each site was matched with a dose gradient across three consistent subjects. After a 21-day expression period, the pigeon brains were harvested for fluorescence analysis. One-way ANOVA was utilized to assess differences in fluorescence intensity, transduction scope, and diffusion range among the different dose gradients within the same brain region.

## Results

### Injection sets verification

In this study, we selected four representative sites for AAV vector injections: the Archistriatum nucleus in the telencephalon, the Nucleus lateralis hypothalami in the diencephalon, the Formatio reticularis medialis mesencephali in the mesencephalon, and the Hippocampus in the pallium. To confirm accurate targeting, the resulting fluorescent brain sections were systematically compared with a standard brain atlas (Fig. S2). Although slight positional deviations within the same nucleus occurred across experimental batches, the overall error was consistently less than 0.5 mm. Only fluorescent sections with injection locis precisely located within the designated brain regions were included in the subsequent analyses.

### Fluorescence analysis validation

To quantitatively analyze fluorescence transduction, we generated heat maps for each brain slice. Each fluorescent image was normalized to a 1700 × 2200 pixel matrix, corresponding to a 170 mm × 220 mm standard pigeon brain map. Analysis revealed that the maximum fluorescence intensity for eGFP was 155, while that for mCherry was 76, reflecting the inherent differences in these fluorescent proteins. We hypothesized that full transduction was achieved when eGFP intensity reached 149, and mCherry reached 76. To validate the analytical accuracy of our approach, we compared the results obtained from our model with those from the widely used ImageJ software (Figure S3A and B). The comparison indicated no significant differences between the two methods (Figure S3C), confirming the reliability of our quantitative analysis.

### Optimum serotype and promoter for anterograde and reanterograde

**Fig. 2:**
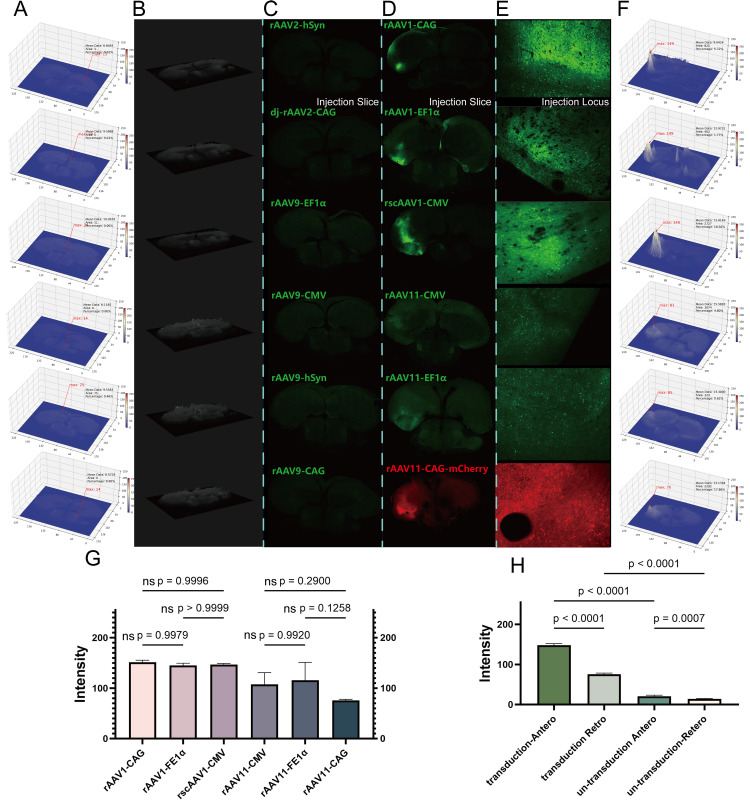
The transduction ability of candidate virus. A) Heat map analysis for un-transduction vectors at injection brain slices; B) VTK pixel perspective for un-transduction vectors at injection brain slices; C) Un-transduction vectors injection brain slices; D) Positive transduction vectors injection brain slices; E) Positive transduction vectors injection locis; F) Heat map analysis for positive transduction vectors at injection brain slices; G) Statistical analysis the significance difference between individual positive transduction vectors; H) Statistical analysis the significance difference of inter-group.

We employed broad-spectrum promoters, including CMV, CAG, and EF1α, as well as the neuron-specific promoter hSyn, and tested serotypes 1, 2, 9, DJ, and 11 to evaluate their transduction capabilities (Figure S1A and B). Positive transduction was defined as fluorescence intensity exceeding a baseline of 30 under the VTK pixel scale (0–255), with visible neuron soma morphology on high-magnification images. Heat map analysis revealed that serotypes 2 and 9, which are commonly used in rodent neurobiology, did not produce detectable expression in the pigeon brain (Fig. 2A–C), regardless of whether they were paired with broad-spectrum or neuron-specific promoters. Similarly, the DJ serotype, previously reported in other studies, showed no positive transduction. In contrast, serotypes 1 and 11 paired with broad-spectrum promoters demonstrated clear expression (Fig. 2D–F). These results suggest that the capsid proteins of serotypes 1 and 11 are more effective at evading the avian immune response and penetrating brain tissue.Analysis of promoter effects indicated no significant differences in expression intensity among CMV, CAG, and EF1α when paired with the same serotype. Within the telencephalon, transduction intensity did not differ significantly between serotypes 1 and 11 (Fig. 2G), although rAAV11-CAG-mCherry exhibited slightly lower intensity, likely due to the intrinsic properties of the mCherry fluorescent protein. As expected, AAV vectors introduced into specific brain sites exhibited distinct transport features: anterograde uptake by the soma with efferent axonal transport, and retrograde uptake by axons with afferent transport to the soma. Notably, serotype 1 also demonstrated limited trans-synaptic transduction at higher doses. Consequently, fluorescence intensity of rscAAV1-CMV-eGFP and rAAV11-EF1α-eGFP varied between injection locis across groups (Fig. 2G and H), while intra-group differences were minimal.Taken together, rscAAV1-CMV-eGFP and rAAV11-EF1α-eGFP were identified as the optimal anterograde and retrograde viral vectors, respectively, and were selected for further testing. For simplicity, rscAAV1-CMV-eGFP and rAAV11-EF1α-eGFP are hereafter abbreviated as AAV1 and AAV11, respectively.

### Transduction efficiency: Cerebral regions

In this study, rscAAV1-CMV-eGFP and rAAV11-EF1α-eGFP were injected into four brain regions: the Hippocampus (Hp), Archistriatum intermedium (AI), Nucleus lateralis hypothalami (LHy), and Formatio reticularis medialis mesencephalic (FRM). Specifically, rscAAV1-CMV-eGFP was delivered to Hp, AI, LHy, and FRM, while rAAV11-EF1α-eGFP was administered to AI, LHy, and FRM. Transduction efficiency and diffusion were evaluated at each injection locus and quantified as the percentage of transduced area in the corresponding brain slices. Transduction intensity in pigeons was also compared with that in mice in the telencephalon, showing no significant differences (Figure S5). For rscAAV1-CMV-eGFP, transduction efficiency at injection locis was comparable between the telencephalon and the Hp, but significantly lower in the diencephalon and mesencephalon (*p < 0.0001*; Figure S4A–C, H). The diffusion range was greatest in the telencephalon (AI) and significantly more restricted in other regions *(p < 0.0001*; Figure S4I). In the Hp, although transduction intensity was high, diffusion was limited due to anatomical constraints imposed by the dura and ventricles. Reduced transduction efficiency and diffusion were also observed in the mesencephalon and diencephalon, likely reflecting differences in neuron soma size and regional anatomy (Figure S4D). For rAAV11-EF1α-eGFP, overall transduction efficiency was lower than that of AAV1, consistent with the use of single-stranded DNA in AAV11 versus double-stranded rscAAV1, which enhances expression speed and efficiency (Figure S4E–G, J). Transduction efficiency varied significantly among the telencephalon, diencephalon, and mesencephalon, with the highest levels observed in the telencephalon and the lowest in the mesencephalon (Figure S4K). Similarly, the diffusion range was greatest in the telencephalon, whereas the diencephalon and mesencephalon showed more restricted spread, reflecting the combined effects of capsid properties and viral tracing characteristics. Collectively, these results demonstrate region-specific differences in transduction efficiency and diffusion for both AAV1 and AAV11, highlighting the importance of brain-region-specific optimization for avian viral vector applications.

### Transduction efficiency: Time

In this chapter, we used the consist dose as 1 μl and setup the time gradient as 21, 30, and 42 Days Post Injection(d.p.i) as transduction period, to test the transduction abilities of rscAAV1-CMV-eGFP and rAAV11-EF1α-eGFP at the telencephalon (AI) and the mesencephalon (FRM) respectively. We conducted the one-way ANOVA to analyze the data across three dimensions: fluorescence intensity, transduction scope, and diffusion range. This analysis aimed to determine the optimal transduction periods for the anterograde AAV1 in both the AI and FRM, as well as for the retrograde AAV11 in the same sites.

**Fig. 3:**
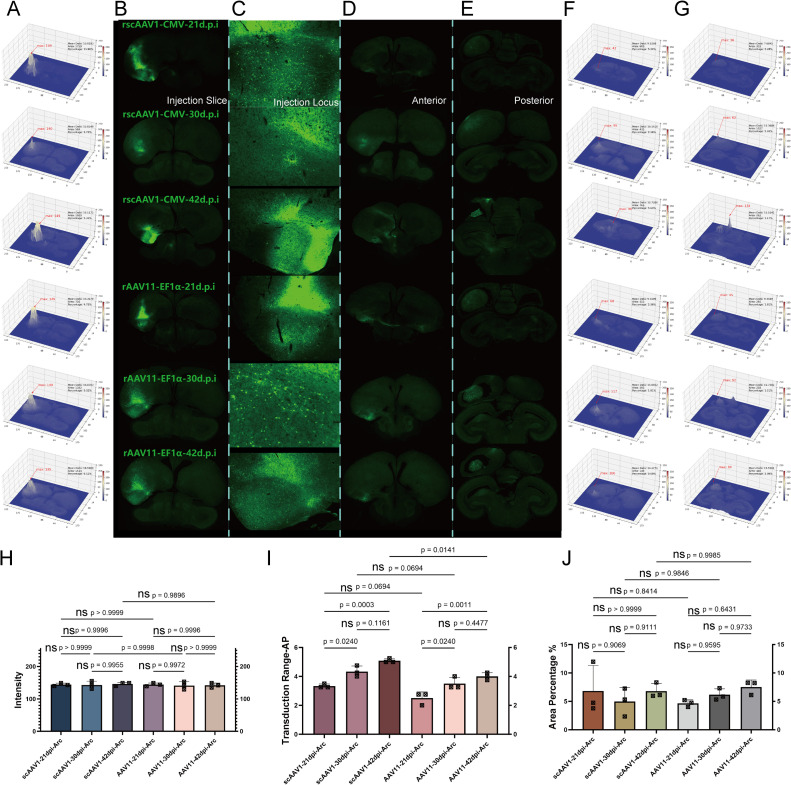
Time gradient test for telencephalon. Transduction ability for each time gradient in AAV1 and AAV11 A) Injection brain slices heat map analysis; B) Injection brain slices; C) Injection locus; D) Transduction range anterior; E) Transduction range posterior; F) Transduction range anterior heat map; G) Transduction range posterior heat map; H) Intensity significance analysis of each tests; I) Transduction scope significance analysis of each tests; J) Expression diffusion range significance of each tests.

In the telencephalon, as shown in the figures with the coronal plane, we displayed the injected brain sections(Fig. 3A and B), injection locis(Fig. 3C), and diffusion range (Fig. 3D anterior and E posterior perspective), and analyzed their fluorescence intensity and diffusion area using VTK(Fig. 3F and G). We concluded as: 1) For fluorescence intensity, there were no significant intra-group differences between AAV1 and AAV11, nor were there inter-group differences, demonstrating that both AAV1 and AAV11 can fully transduce within the telencephalon over a 21-day period(Fig. 3H). 2) For transduction scope, AAV1 and AAV11 showed a common trend within groups; differences between 42 days and 30 days were not significant, while differences between 30 days and 21 days were significant. There were no significant differences between groups at the same time gradients, indicating that a sufficient transduction scope had been achieved by day 21 and had become saturated by day 30(Fig. 3I). 3) For diffusion range, calculations showed no significant intra-group or inter-group differences for AAV1 and AAV11. The minimum diffusion range reached up to 4.7 %, fully covering and partially leaking beyond the targeted brain area(Fig. 3J). Thus, in the telencephalon, increasing the transduction period to enhance efficiency is not necessary. Our analysis of the fluorescence brain sections of rscAAV1-CMV-eGFP and rAAV11-EF1α-eGFP across the time gradient in these three dimensions concluded that the recommended optimal transduction period is 21 days.

**Fig. 4:**
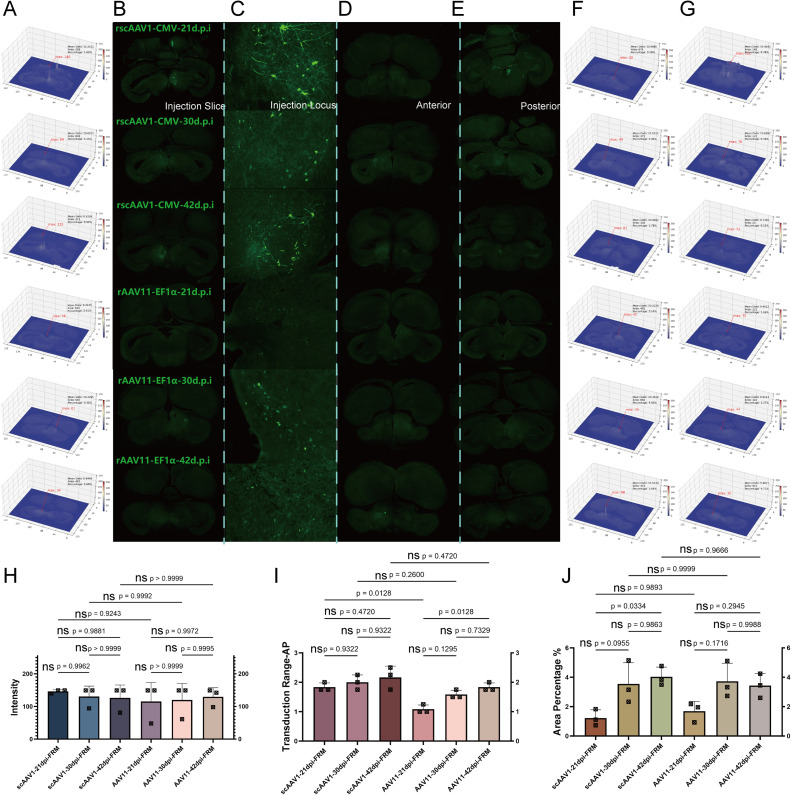
Time gradient test for mesencephalon. Transduction ability for each time gradient in AAV1 and AAV11 A) Injection brain slice heat map analysis; B) Injection brain slice; C) Injection locus; D) Transduction range anterior; E) Transduction range posterior; F) Transduction range anterior heat map; G) Transduction range posterior heat map; H) Intensity significance analysis of each tests; I) Transduction scope significance analysis of each tests; J) Expression diffusion range significance of each tests.

In the mesencephalon region, as illustrated, we displayed the injection brain slices(Fig. 4A and B), injection locis(Fig. 4C), and diffusion range (Fig. 4D anterior and E posterior) on the coronal plane, and analyzed their fluorescence intensity and diffusion area using VTK(Fig. 4F and G). 1) Regarding fluorescence intensity, there were no significant intra-group differences between AAV1 and AAV11, nor were there significant inter-group differences, indicating that both AAV1 and AAV11 achieved sufficient transduction in the mesencephalon within a 21-day period(Fig. 4H). 2) For transduction scope, the differences among these three time gradients for AAV1 were not significant, while for AAV11 there was a significant difference between 42 days and 21 days, with a significant difference at the 21-day transduction period when comparing inter-group. This indicates that AAV1 achieved a sufficient transduction scope by 21 days, whereas AAV11 had not yet reached sufficient transduction(Fig. 4I). 3) In diffusion range, significant differences were found between 21 and 42 days for AAV1, but intra-group differences for AAV11 were not significant, with overall inter-group differences also not significant. The minimal diffusion range reached up to 1.4 %, covering the almost target brain area(Fig. 4J). The overall limited diffusion ability in this region may be related to differences in the type of neuronal soma. Therefore, in the mesencephalon region, increasing the transduction period to enhance efficiency is not necessary. After analyzing the fluorescence brain slices of rscAAV1-CMV-eGFP and rAAV11-EF1α-eGFP across three dimensions under different time gradients, we recommend a 21-day period as the optimal transduction period for the mesencephalon.

Overall, both in the telencephalon and mesencephalon regions, the duration of transduction does not significantly impact overall transduction efficiency. Although AAV11 in the mesencephalon shows slightly insufficient transduction with a smaller overall transduction scope, we speculated this serotype may present toxicity for pigeons, causing some individuals not to survive until 21 days post-injection. Therefore, it is not advisable to extend the transduction period to increase efficiency. Based on this situation, the transduction period for both viruses in the mesencephalon and telencephalon was set as 21 days, and for further dose gradient experiments.

### Transduction efficiency: Dose

In this chapter, we designed dose gradients of 0.5 μl, 1 μl, and 2 μl to test the transduction capabilities of rscAAV1-CMV-eGFP and rAAV11-EF1α-eGFP in the telencephalon at the AI site and in the mesencephalon at the FRM site. One-way ANOVA analysis was conducted across three dimensions: fluorescence intensity, transduction scope, and diffusion range. This analysis aimed to determine the optimal doses for anterograde transduction by AAV1 in both the AI of the telencephalon and the FRM of the mesencephalon, as well as for retrograde transduction by AAV11 in the same regions.

**Fig. 5:**
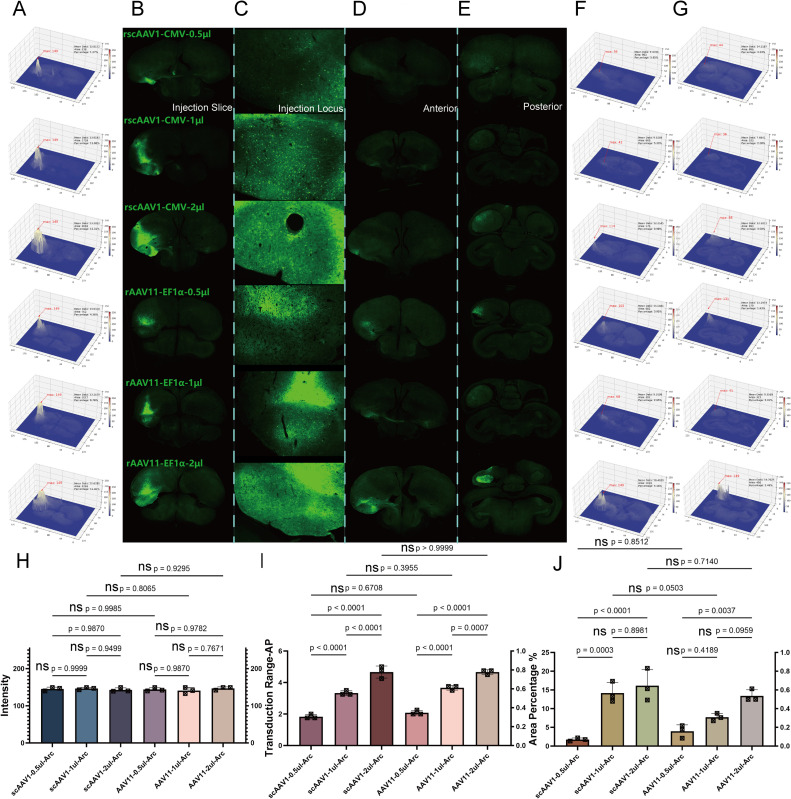
Dose gradient test for telencephalon. Transduction ability for each dose gradient in AAV1 and AAV11 A) Injection brain slice heat map analysis; B) Injection brain slices; C) Injection locus; D) Transduction range anterior; E) Transduction range posterior; F) Transduction range anterior heat map; G) Transduction range posterior heat map; H) Intensity significance analysis of each tests; I) Transduction scope significance analysis of each tests; J) Expression diffusion range significance of each tests.

In the telencephalic region, as shown in the coronal views, we displayed injection brain slices(Fig. 5A and B), injection locis(Fig. 5C), and the range of diffusion (Fig. 5D anterior and E posterior), and analyzed their fluorescence intensity and diffusion range using VTK(Fig. 5F and G). 1) For fluorescence intensity, there were no significant intra-group differences between AAV1 and AAV11, nor were there significant inter-group differences, demonstrating that both AAVs could fully transduction within the telencephalon at a dose of 0.5 μl(Fig. 5H). 2) For transduction scope, significant differences were observed between the three dose gradients for both AAV1 and AAV11, while inter-group comparisons showed no significant differences. This indicates that the range of transduction scope significantly increases with the rise in dose gradient. AAV1 and AAV11 displayed similar transduction capabilities within the telencephalion(Fig. 5I). 3) For diffusion range, significant differences were found between 0.5 μl and 2 μl dosages for both AAV1 and AAV11, with no significant overall inter-group differences. The minimal diffusion range as 4.3 %, covering and leaking beyond the targeted brain area (Fig. 5J). Therefore, in the telencephalion region, both AAV1 and AAV11 achieve effective transduction with the lowest injection dose. After analyzing the fluorescence brain slices of rscAAV1-CMV-eGFP and rAAV11-EF1α-eGFP across the three dimensions under dose gradients, we concluded that the optimal dosage for mesencephalic transduction is 0.5 μl for both vectors.

**Fig. 6:**
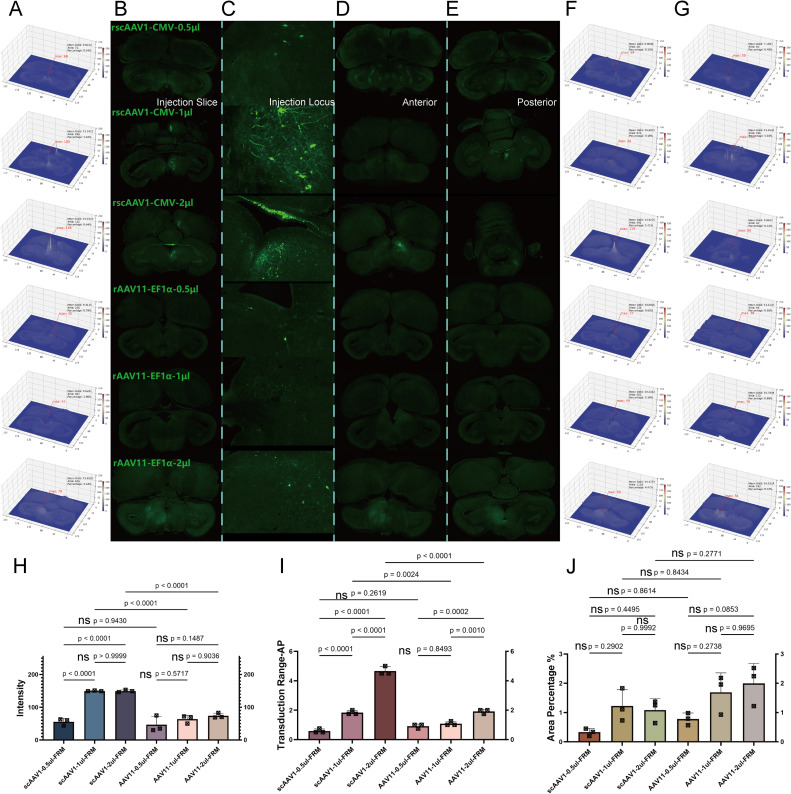
Dose gradient test for mesencephalon. Transduction ability for each dose gradient in AAV1 and AAV11 A) Injection brain slices heat map analysis; B) Injection brain slices; C) Injection locus; D) Transduction range anterior; E) Transduction range posterior; F) Transduction range anterior heat map; G) Transduction range posterior heat map; H) Intensity significance analysis of each tests; I) Transduction scope significance analysis of each tests; J) Expression diffusion range significance of each tests.

In the mesencephalic region, as depicted in the coronal sections, we displayed injection brain slices(Fig. 6A and B), injection locis(Fig. 6C), and the range of diffusion (Fig. 6D anterior and E posterior), and analyzed their fluorescence intensity and diffusion area using VTK(Fig. 6F and G). 1) For fluorescence intensity, intra-group differences within the AAV11 group were not significant. In AAV1, significant differences were observed between 0.5 μl, 1 μl, and 2 μl, and except for the 0.5 μl dose, inter-group differences were significant. This demonstrates that dose significantly affects the transduction efficiency of AAV1, while for AAV11, although there is an increased the trend were not significant(Fig. 6H). 2) For transduction scope, significant differences were observed between the three dose gradients for AAV1. In AAV11, significant differences were found between 2 μl and, both 1 μl and 0.5 μl, with significant differences in inter-group comparisons except at 0.5 μl. The rise in dose gradient had a lesser effect on the sagittal direction transduction of AAV11 compared with AAV1(Fig. 6I). 3) For diffusion range, intra-group differences for both AAV1 and AAV11 were not significant, and overall inter-group differences were also not significant. The diffusion range was less and under 2 %, covering parts of the target brain area. The limited overall diffusion range in this area may be related to differences in neuronal soma types(Fig. 6J). Therefore, in the mesencephalic region, enhancing transduction efficiency can be achieved by appropriately increasing the injection dose. After analyzing the fluorescence brain slices of rscAAV1-CMV-eGFP and rAAV11-EF1α-eGFP across three dimensions under time gradients, we recommend the optimal injection dosages in the mesencephalon to be 1 μl for AAV1 and 2 μl for AAV11.

Here, we could conclude that within the telencephalon, as the injection dose elevated, the transduction capabilities of AAV11 and AAV1 show similar raise patterns, with no significant differences between groups. In the mesencephalon, however, as the dose gradient increases, significant differences emerge in the transduction efficiency and the range of sagittal direction transduction for AAV11 compared with AAV1. This is partly due to, in this area with a reticular structure, retrograde AAV11 was transported by axons and delivered to the soma, hence the area's brightness mainly decline and only reflected by successfully transduction axons. On contrary, anterograde AAV1 transduction is soma-directed and its accumulation was significantly greater than retrograde. Additionally, due to the positional characteristics of this area, a high dose of anterograde AAV1 can continue to propagate down the reticulospinal tract, resulting in a transduction range that is significantly greater than that of retrograde AAV11. Based on this situation, the injection dose for both viruses was set at 0.5 μl in the telencephalon, while in the mesencephalon it was set at 1 μl for AAV1 and 2 μl for AAV11.

### Optogenetics components test

Currently, it is true that much work in avian research uses optogenetics technology and has achieved some positive results. However, these studies are mostly related to visual and auditory functions rather than locomotion behavior. Additionally, the mesencephalon remains a blind spot for these studies, which are more focused on the telencephalon. Consequentially, we tested the expression capabilities of AAV vectors connection optogenetic components combination with broad-spectrum promoters, using the recommended expression times and doses concluded above. Due to the differences in the types of neurons in the telencephalon and mesencephalon, we conducted separate transduction tests for each region.Therefore, we assessed the transduction abilities of serotype 1 and 11 AAVs combined with the activated component hChR2, and the Cre/Dio strategy, as well as the suppressive component eNpHR3.0 in both the telencephalon and mesencephalon, respectively(Figure S6 A - C and E). Sequentially the result indicated, the same serotype and promoter in viruses, with or without the hChR2 component(Figure S7), show significant differences in transductability between the mesencephalon and telencephalon. And numerous experiments to test the transduction capacity AAVs vectors accompany with hChR2 component, unfortunately, we have not achieved any positive results in mesencephalon but positively in telencephalon, whether using AAV 1 serotype and 11 serotype, both of which transduce well with broad-spectrum promoters connected to eGFP components directly in these brain locations. Furthermore, we also tested the Cre/DIO strategy as used in mice, AAV1-CMV-Cre mixed with AAV11-Ef1α-DIO-hChR2-eYFP and injected together, but there was minimal transduction. Fluorescent at the injection slices were analyzed for intensity at the VTK environment, including a VTK pixel perspective display for more details in the mesencephalic slices(Figure S6 D). Intensity results from the telencephalon and mesencephalon were compared with the baseline. In the telencephalon, except for rAAV11-EF1α-hChR2-eGFP, which showed no significant difference from and was higher than the baseline, all others showed significant differences(Figure S6 F). In the mesencephalon, none of the five vectors showed significant differences from the baseline (Figure S6 G), with only rAAV11-GAG-hChR2-eYFP and rAAV11-EF1α-eNpHR3.0-eGFP a little bit exceeding the baseline. This indicates that while the five vectors can achieve desirable transduction in the telencephalon, the results in the mesencephalon were not well.

## Discussion

### The treatment groups arrangement in this work

In this study, the grouping strategy was deliberately structured to address distinct experimental objectives while ensuring comparability across conditions. To establish the fundamental transduction capacity of recombinant AAVs in the pigeon brain, 12 treatment groups comprising 36 valid subjects were dedicated to baseline viral testing. To further investigate location, temporal and dosage effects, 23 additional groups were organized, including 11 groups (32 valid subjects) receiving AAV11 and 12 groups (34 valid subjects) receiving AAV1, thereby enabling a direct comparison of transduction kinetics and dose–response relationships between the two virus. Beyond efficiency testing, 10 groups with 30 valid subjects were designed for optogenetic experiments. To place these findings in a broader context, four comparative groups were included—two in pigeons and two in mice—providing a cross-species reference for viral performance. Finally, one blank control group was incorporated by 1 μl saline injection both to assess potential adverse effects of the surgical procedure itself and to establish baseline fluorescence levels for subsequent quantification.

Together, this multi-tiered experimental design provided a comprehensive framework that not only validated viral transduction efficiency but also systematically addressed serotype-specific parameters, functional applicability, and interspecies comparability. Such an arrangement ensured that the findings were interpretable across multiple dimensions of viral performance, thereby strengthening the reliability and generalizability of the conclusions.

### Optimum proportion: Retro/Antero

In this study, through a series of experiments, we identified AAV vectors capable of successful transduction in the avian brain and determined the optimal methods for different target regions (Table 2). The primary goal of this work was to establish practical and reliable guidelines, both for experimental paradigms and for the use of AAV serotypes. This is particularly valuable for laboratories like ours that may not have extensive molecular biology expertise or the capacity to construct customized vectors or develop viruses home-made. From an experimental perspective, our results demonstrate that in non-model animal studies, researchers can efficiently select from commercially available viruses to achieve effective transduction. By providing recommended strategies for retrograde and anterograde tracing, as well as for avian nucleus co-localization studies, this work offers actionable guidance that enables the rapid initiation and advancement of future research in avian neuroscience.

**Table 2:** The optimize time and dose for each location and each AAV.

Vector	sc-AAV1-CMV-eGFP		AAV11-EF1α-eGFP	
Site	Telencephalon	Mesencephalon	Telencephalon	Mesencephalon
**Time (d.p.i.)**	21	21	21	21
**Dose (μl)**	0.5	1	0.5	2

Although some studies have reported the use of AAV vectors in avian research, these applications primarily employed AAVs as auxiliary tools and focused on specific biological questions—such as songbird cognitive learning or avian visual discrimination—rather than systematically examining viral transduction periods or dosage parameters (Düring et al., 2020; Shimizu et al., 2010). As a result, their findings provide only limited guidance for the practical use of AAVs. Moreover, most of these studies concentrated on viral transduction in telencephalic nuclei, including Area X, Field L, and HVC, while largely neglecting the mesencephalon (Sánchez-Valpuesta et al., 2019; Rook et al., 2021). However, the avian mesencephalon also plays an important role in regulating motor and cognitive behaviors, underscoring the need to develop viral tools capable of probing this region (Grillner and El Manira, 2020; Josset et al., 2018; Cai et al., 2015). In our study, we therefore extended the analysis to the mesencephalon alongside the telencephalon and found substantial differences in transduction efficiency between these two brain regions. Importantly, methods that proved effective in the telencephalon could not be directly applied to the mesencephalon. We hypothesize that these discrepancies may arise from differences in neuronal size, morphology, and intrinsic properties between the two regions, although further empirical investigation is required to validate this explanation.

### Model animals comparison

It is well established that mice and other rodents are the most widely used model animals in neurobiology. Consequently, a broad range of viral vectors has been developed and optimized for different experimental purposes. At the promoter level, options extend beyond ubiquitous promoters such as hSyn (Kügler et al., 2003), which efficiently and specifically drives expression in mature neurons, to neurotransmitter-specific promoters that target distinct neuronal subtypes—for example, the GABAergic promoter hVGAT, the dopaminergic promoter mTH, and the cholinergic promoter ChAT—as well as glial cell–specific promoters such as hNPPC (Kojima et al., 2024; Griffin et al., 2019; Santoscoy et al., 2023; Nieuwenhuis et al., 2021; Zhou et al., 2019).. At the serotype level, AAV1 is known for its stable yet gradual increase in expression, AAV5 exhibits relatively low expression in the liver, and AAV9 demonstrates strong retrograde transport capabilities alongside high expression efficiency. In addition to AAVs, other viral vectors such as rabies virus (RV), pseudorabies virus (PRV), herpes simplex virus (HSV), and vesicular stomatitis virus (VSV) have also been employed to expand the available toolkit (Liu et al., 2022; Li et al., 2019). However, to date, no systematic studies have explored how these promoters, serotypes, or alternative viral vectors might be applied or adapted specifically for avian models. Given the vast scope of this challenge, the present study addresses only a subset of these questions, providing a first step toward the broader development of viral tools for avian neuroscience.

Previous studies in mammalian models have systematically optimized adeno-associated virus (AAV) vectors through the adaptation of serotypes and promoter elements to specific tissues, thereby establishing a robust foundation for rodent neuroscience. For instance, widely used protocols in mice often involve injecting approximately 200 nL of viral suspension per brain region at a rate of 60 nL/min in two-month-old animals, followed by an expression period of about 21 days, though functional experiments can begin as early as 14 days (van der Zouwen et al., 2021; Inagaki et al., 2022; Lee et al., 2020). These parameters have been fine-tuned primarily for the rodent cortex, where circuit mapping, optogenetic manipulations, and behavioral assays are well established. In contrast, direct extrapolation of these approaches to avian species is problematic. Birds differ markedly in brain size, structure, and organization, with the pallium serving functions analogous to the mammalian cortex but lacking fully defined functional boundaries and clear homologies. As a result, viral transduction efficiency in pigeons varies considerably across brain regions, and rodent-derived injection volumes, expression windows, or targeting strategies often fail to yield consistent outcomes. This variability underscores the need for empirical determination of species-specific parameters in avian models. Moreover, while the historical progression of AAV development has expanded from naturally occurring serotypes with narrow tropism to engineered variants with enhanced specificity and efficiency, these advances have largely focused on mammalian systems. Differences in promoter activity, serotype tropism, and neuronal targeting across species limit their applicability to birds. Consequently, certain experimental paradigms that are reliable in rodents—for example, using optogenetic stimulation in the motor cortex to modulate pain or targeting the somatosensory cortex to dissect projection-specific functions—cannot be directly adapted to the avian pallium. Taken together, these considerations highlight a critical gap in viral tool development for birds. Establishing avian-specific strategies—including optimized serotypes, promoters, and regulatory elements—will be essential for enabling robust transduction, accurate circuit mapping, and advanced manipulations in avian neuroscience (Islam et al., 2020; Nguyen et al., 2024). Future work should therefore focus on systematically refining viral vectors in pigeons and related species to build reliable experimental paradigms tailored to the unique features of the avian brain.

### Limitation of this study

In this study, we conducted only a single test in the Hp and LHy regions, rather than performing systematic time-course and dosage-gradient analyses as we did for the telencephalic AI and mesencephalic FRM. This decision was based on two considerations. First, although the Hp was historically regarded as part of the avian cortex, it is now classified as a subdivision of the telencephalon under the updated brain nomenclature. In our experiments, transduction intensity in the Hp was comparable to that observed in the AI, with the main differences in diffusion range attributable to physical constraints imposed by the dura and ventricular boundaries. Given the Hp’s positional and functional relevance, we did not consider retrograde tracing necessary in this region and therefore did not test AAV11. Second, the LHy’s anatomical location, being surrounded by ventricles, presented substantial technical challenges. As a result, complete datasets could not be obtained from three subjects. Taken together, these factors explain why comprehensive transduction analyses were not pursued in the Hp and LHy within the current study.

In this experiment, the viral vectors tested did not encompass every possible scenario. For example, we evaluated more serotypes with retrograde properties than with anterograde ones; we did not perform a strict comparison of transduction efficiencies among the CMV, CAG, and EF1α promoters for each serotype; AAV1 was tested in a double-stranded DNA form, whereas AAV11 was tested as a single-stranded DNA vector; and AAV1 employed the CMV promoter while AAV11 used EF1α. These variations primarily arose because all viral vectors were sourced from a domestic biotechnology company, and thus our selection was constrained by the available stock and by vectors that could be rapidly generated from existing plasmids. Despite these limitations, the study nevertheless provides valuable insights: the results capture both anterograde and retrograde transduction characteristics and establish practical reference points for optimal injection doses and timing across different brain regions.

## Conclusion

Through careful experimental design and systematic evaluation, this study assessed the expression efficiency of multiple viral vectors across different injection locis and established specific recommendations for their optimal use. Based on our findings, we recommend employing serotype 1 in combination with a CMV promoter (rscAAV1-CMV-eGFP) for anterograde tracing and serotype 11 with an EF1α promoter (rAAV11-EF1α-eGFP) for retrograde tracing. The most effective transduction period was observed at 21 days post-injection. For the telencephalon, we recommend a dosage of 0.5 μl for both vectors, whereas in the mesencephalon, optimal doses were 1 μl for AAV1 and 2 μl for AAV11. In addition, we evaluated the potential of these vectors for optogenetic applications, finding that while both regions supported expression, the mesencephalon exhibited greater limitations compared with the telencephalon. Collectively, these results provide practical guidance for avian neuroscience, offering optimized parameters for viral tracing and functional manipulation. We anticipate that this framework will facilitate future research into neural circuits in birds and contribute to the broader advancement of avian neurobiology.

## CRediT authorship contribution statement

**Zhengyue Zhou:** Writing – original draft, Investigation, Formal analysis, Data curation. **Yezhong Tang:** Writing – review & editing, Methodology. **Wenbo Wang:** Project administration, Funding acquisition. **Xin Yang:** Resources, Data curation. **Zihan Zhuang:** Visualization, Formal analysis, Data curation. **Tianmei Pu:** Validation, Conceptualization. **Feng Jiang:** Supervision. **Zhendong Dai:** Supervision, Funding acquisition.

## Disclosures

The authors declare no conflicts of interest.

In the process of writing the article, all authors invested lots of time and energy. Among the authors in the list, WenboWang and ZhendongDai designed research; Zhengyue Zhou performed research; Zihan Zhuang and Tianmei Pu analyzed and interpreted the data; Zhengyue Zhou and Yezhong Tang wrote and edited the paper. Xin Yang provide the mice data.

## Appendix Supplementary materials

Image, application 1
