# Supplementary material for: Optimization of virus-mediated functional imaging based on quantitative evaluation of transduction efficiency in pigeon (Columba livia domestica)
Source: Poult Sci. 2025 Oct 8;104(12):105961. doi: 10.1016/j.psj.2025.105961 (PMC12593608; doi:10.1016/j.psj.2025.105961)
Supplement: Supplementary file 1 [file mmc1.docx]

# Supplementary material


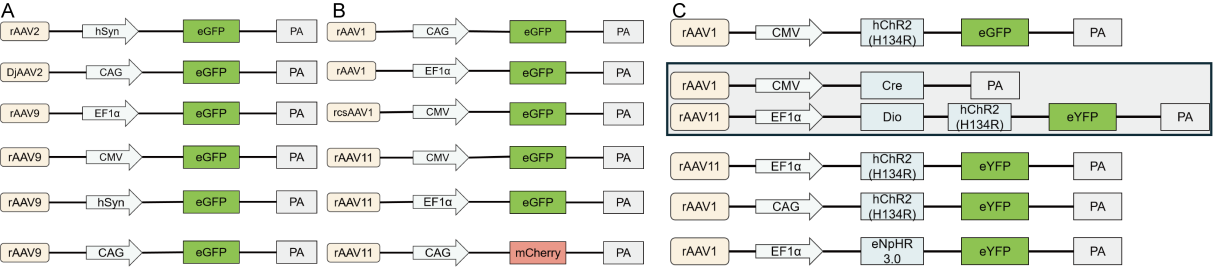


Figure S1 All vectors tested in this work, related to Figure2.

A) Un-transduction vectors;

B) Succesfully tranduction vectors;

C) optogenetic tested vectors.


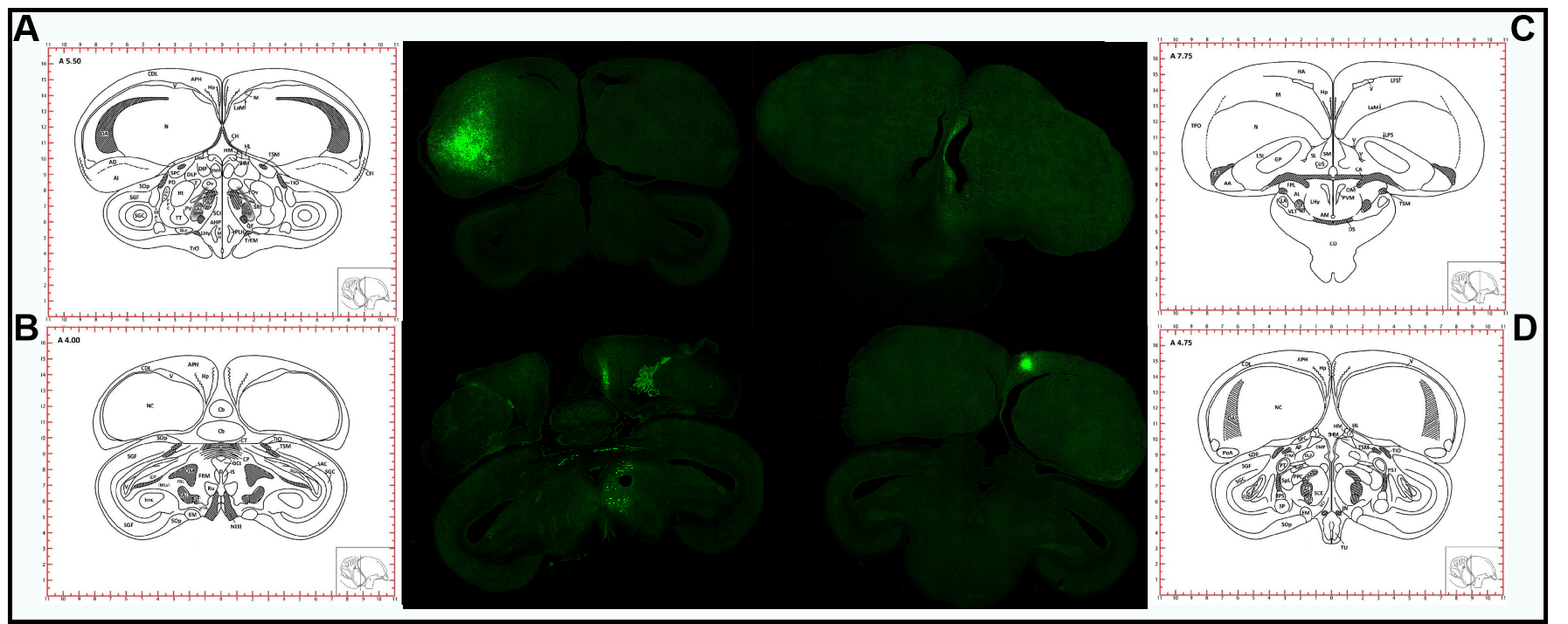


Figure S2 Injection slice compared with pigeon standard brain map.

A) AI; B) FRM; C) LHy; D) Hp.


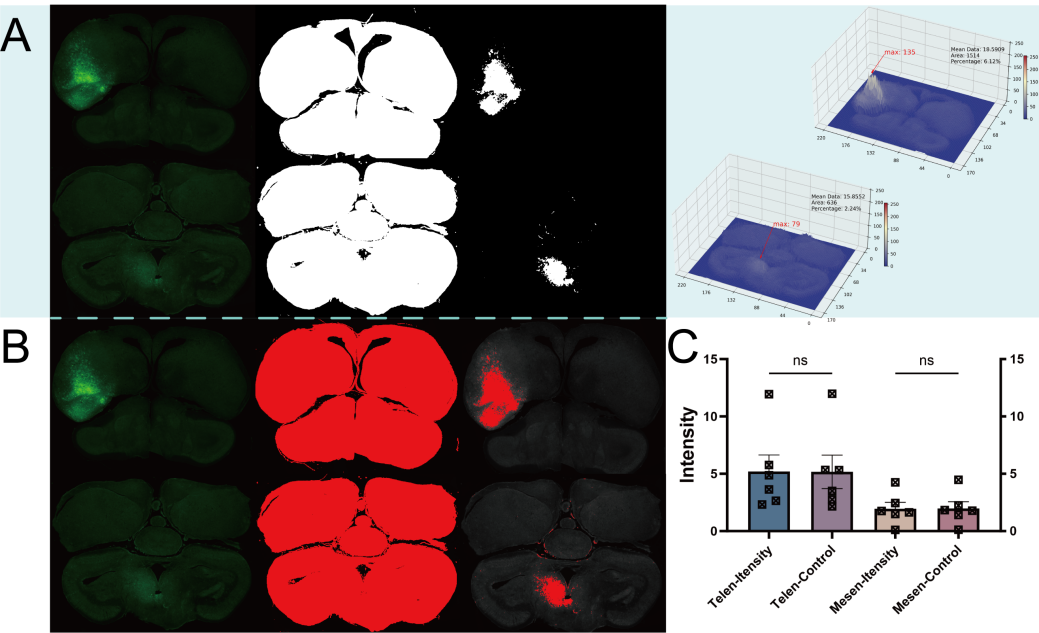


Figure S3 VTK analysis compared with Image-J analysis.

A) VTK analysis;

B) Image-J analysis;

C) Statistical analysis significance of fluorescence intensity of two methods.


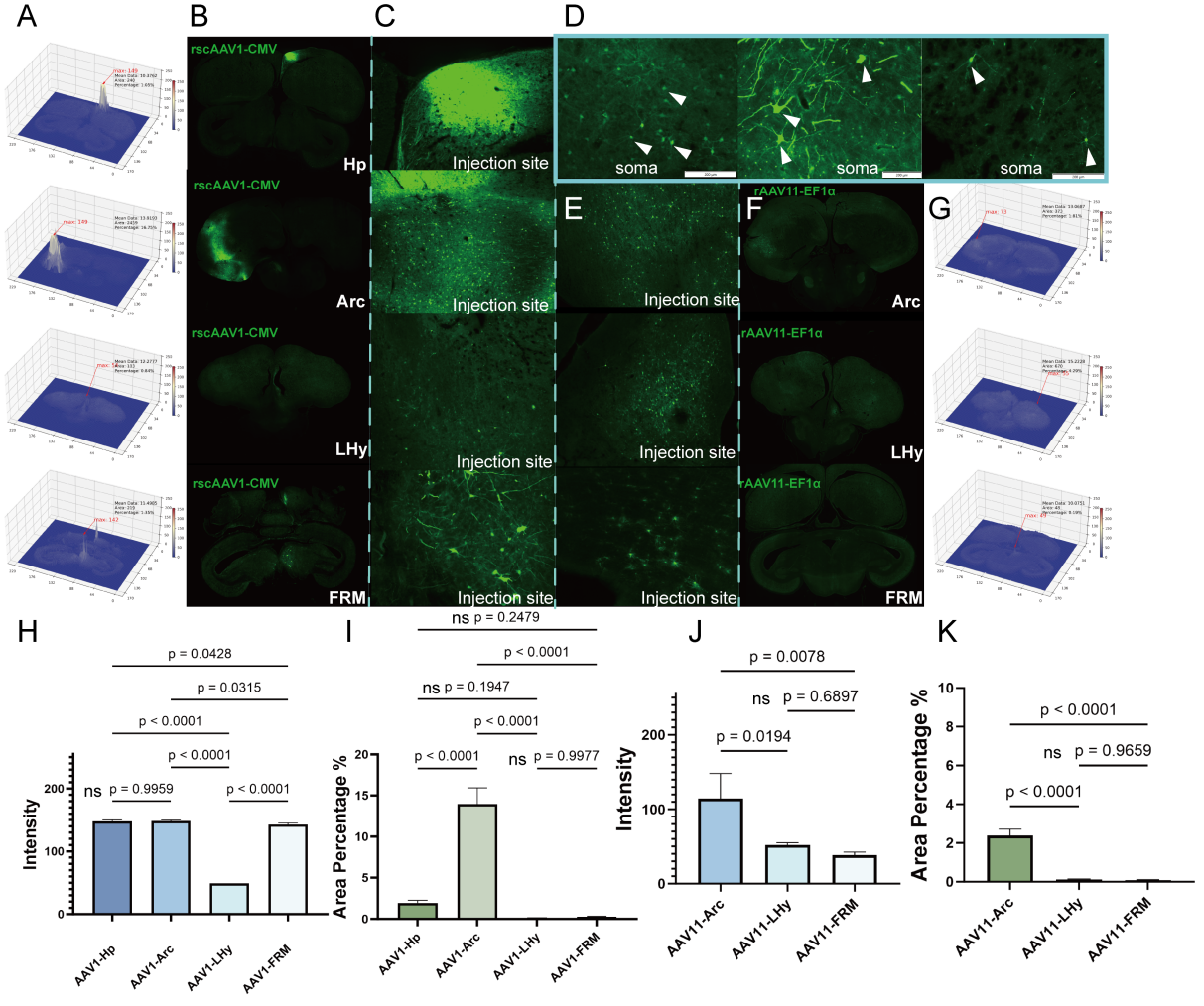


Figure S4. Transduction test of vector AAVs in different cerebral region.

A) Heat map analysis for AAV1 transduction in Hp, AI, LHy and FRM;

B) Injection and transduction brain slices of AAV1;

C) Injection and transduction locus of AAV1;

D) Different features of soma;

E) Injection and transduction brain slices of AAV11;

F) Injection and transduction locis of AAV11;

G) Heat map analysis for AAV11 transduction in AI, LHy and FRM;

H) Statistical analysis significance of AAV1 transduction intensity;

I) Statistical analysis significance of AAV1 transduction scope;

J) Statistical analysis significance of AAV11 transduction intensity;

K) Statistical analysis significance of AAV11 transduction scope.


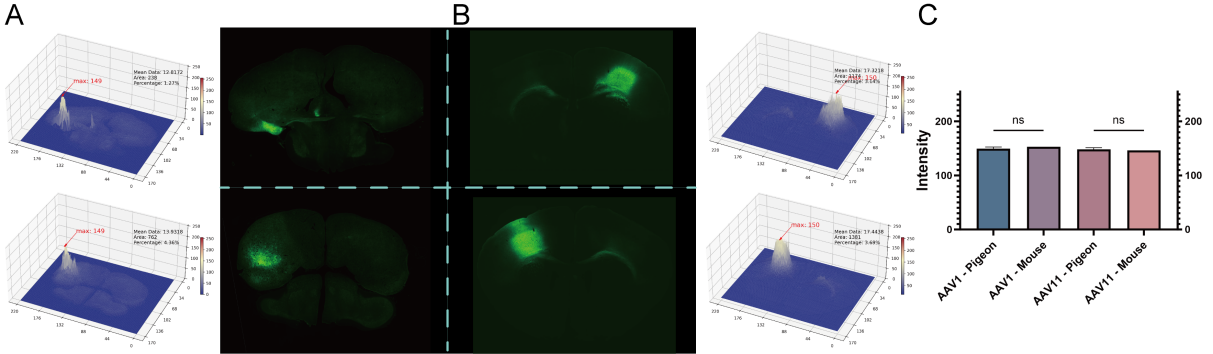


Figure S5 The transduction ability compared pigeons with mice in telencephalon. (The mice brain fluorescence slice were applied by Brain Case, and obtained the usage license from it.)

A) The transduction result of guideline for pigeons, AAV1(above) and AAV11(below);

B) The transduction result of guideline for mice, AAV1(above) and AAV11(below);

C) Significance analysis the fluorescence intensity of two animals.


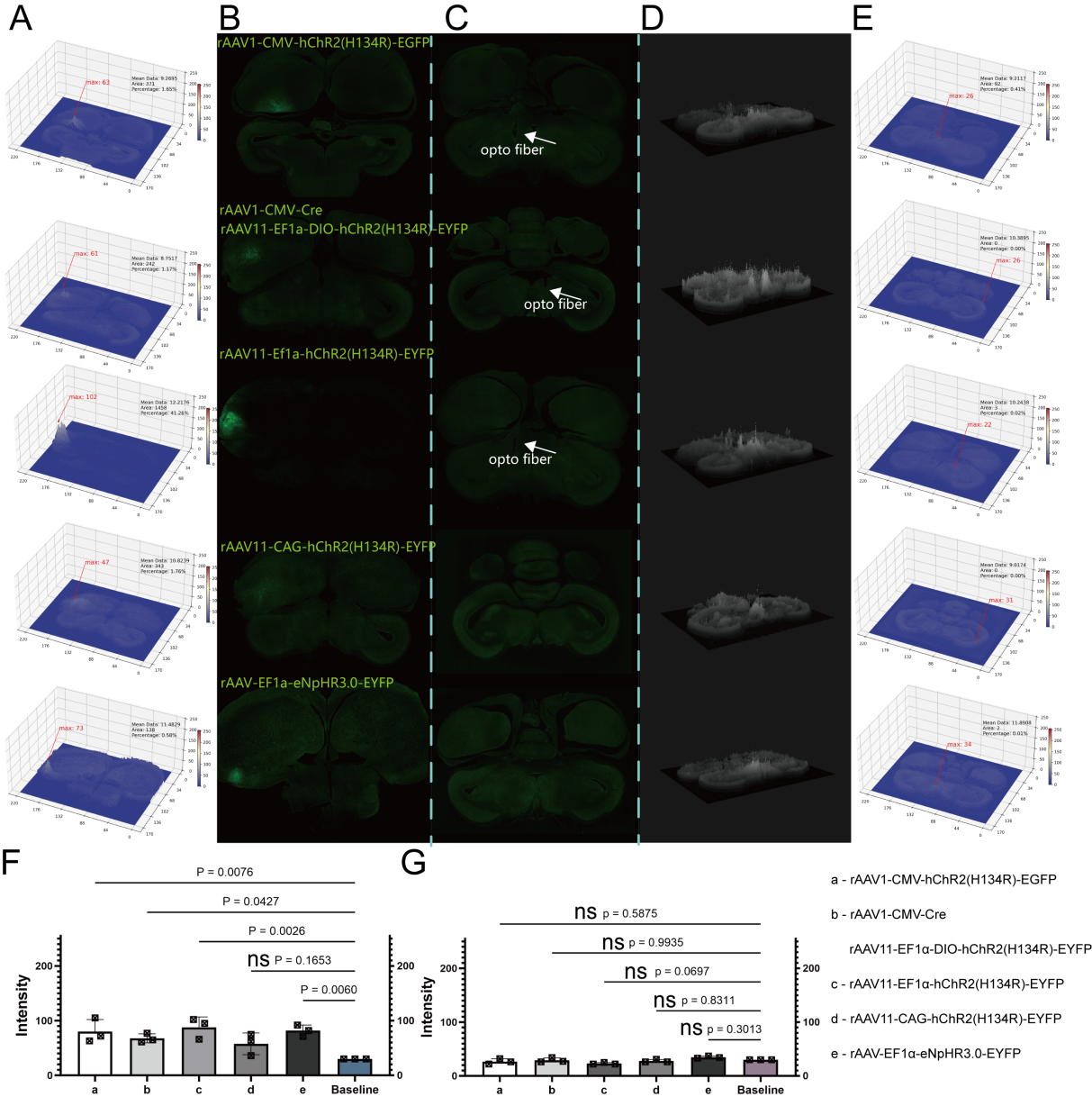


Figure S6 Optogenetics components test. Transduction ability for each optogenetic vectors:

A) Telencephalon virus injection slice heat map;

B) Telencephalon virus injection slices;

C) Mesencephalon virus injection slices;

D) Mesencephalon virus injection VTK analysis;

E) Mesencephalon virus injection slice heat map;

F) Telencephalon intra-group intensity significance comparison;

G) Mesencephalon intra-group intensity significance comparison.


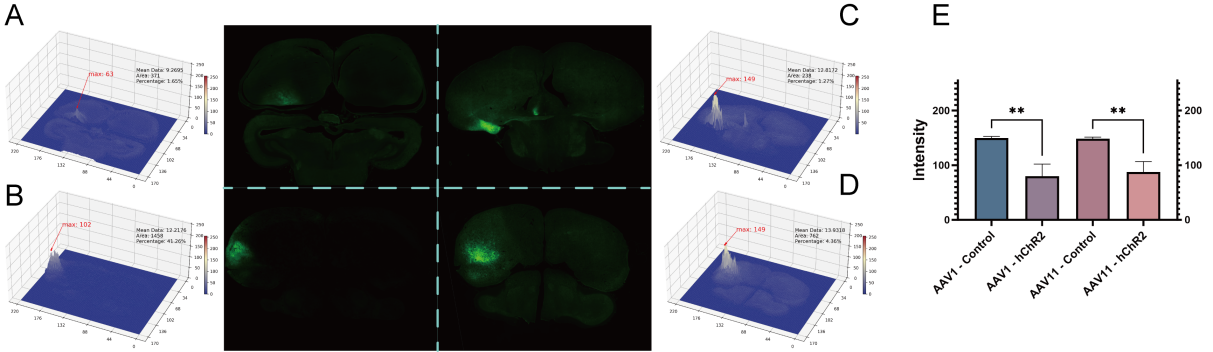


Figure S7 The transduction ability in telencephalon with and without hChR2 component.

1. The transduction result of control for pigeons, AAV1;
2. The transduction result of hChR2 component for pigeons, AAV1;
3. The transduction result of control for pigeons, AAV11;
4. The transduction result of hChR2 component for pigeons, AAV11;
5. Statistical analysis significance of fluorescence intensity between with and without hChR2 component


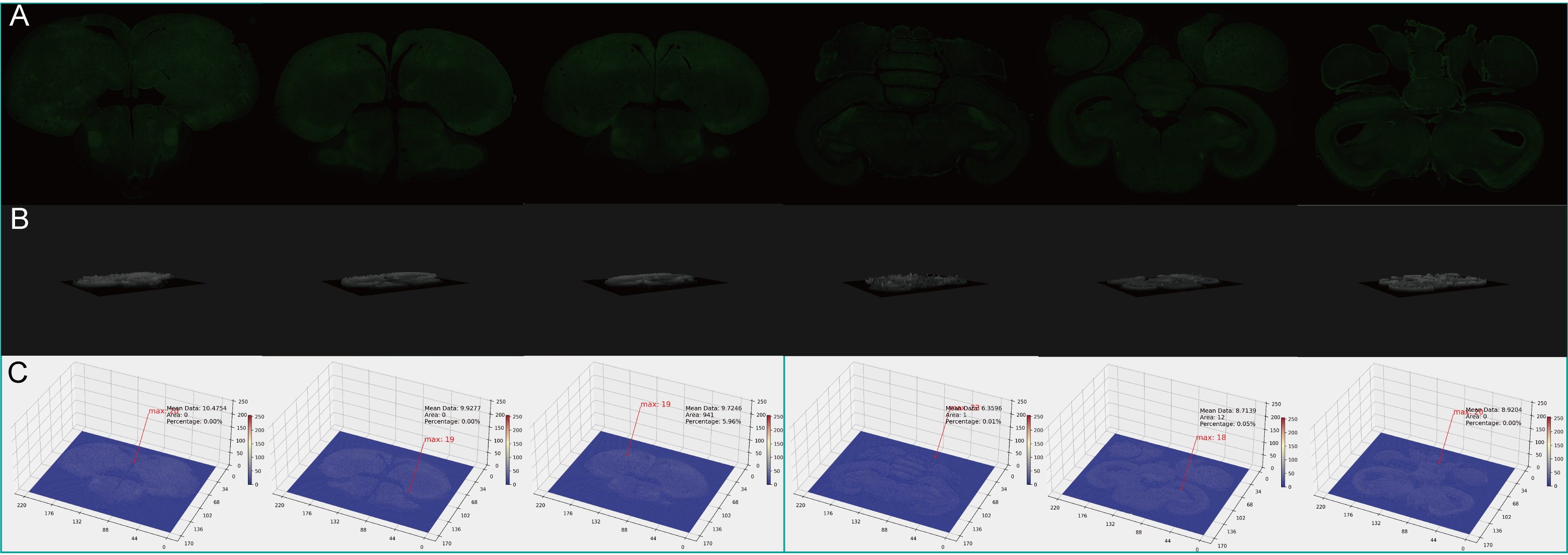


Figure S8 Blank control experiments

1. The the brain slice processed by saline injection;
2. The intensity analysis under the VTK pixel perspective;
3. The intensity analysis under the heat map perspective;
